# Duchenne muscular dystrophy gene expression is an independent prognostic marker for IDH mutant low-grade glioma

**DOI:** 10.1038/s41598-022-07223-2

**Published:** 2022-02-25

**Authors:** Michael Naidoo, Leanne Jones, Benjamin Conboy, Wael Hamarneh, Darwin D’Souza, Karen Anthony, Lee R. Machado

**Affiliations:** 1 Centre for Physical Activity and Life Sciences, University of Northampton, University Drive, Northampton, NN1 5PH UK; 2 Northampton General Hospital NHS Trust, Northampton, NN1 5BD UK; 3 Icahn School of Medicine at Mount Sinai, New York City, USA; 4 Department of Genetics and Genome Science, University of Leicester, Leicester, LE1 7RH UK

**Keywords:** CNS cancer, Cancer, Molecular biology, Neuroscience, Diseases of the nervous system, Glial biology, Molecular neuroscience

## Abstract

Alterations in the expression of the Duchenne muscular dystrophy (DMD) gene have been associated with the development, progression and survival outcomes of numerous cancers including tumours of the central nervous system. We undertook a detailed bioinformatic analysis of low-grade glioma (LGG) bulk RNAseq data to characterise the association between *DMD* expression and LGG survival outcomes. High *DMD* expression was significantly associated with poor survival in LGG with a difference in median overall survival between high and low *DMD* groups of over 7 years (P = < 0.0001). In a multivariate model, *DMD* expression remained significant (P = 0.02) and was an independent prognostic marker for LGG. The effect of *DMD* expression on overall survival was only apparent in isocitrate dehydrogenase (IDH) mutant cases where non-1p/19q co-deleted LGG patients could be further stratified into high/low *DMD* groups. Patients in the high *DMD* group had a median overall survival time almost halve that of the low *DMD* group. The expression of the individual *DMD* gene products Dp71, Dp71ab and Dp427m were also significantly associated with overall survival in LGG which have differential biological effects relevant to the pathogenesis of LGG. Differential gene expression and pathway analysis identifies dysregulated biological processes relating to ribosome biogenesis, synaptic signalling, neurodevelopment, morphogenesis and immune pathways. Genes spanning almost the entirety of chromosome 1p are upregulated in patients with high overall *DMD*, Dp71 and Dp427m expression which worsens survival outcomes for these patients. We confirmed dystrophin protein is variably expressed in LGG tumour tissue by immunohistochemistry and, overall, demonstrate that *DMD* expression has potential utility as an independent prognostic marker which can further stratify IDH mutant LGG to identify those at risk of poor survival. This knowledge may improve risk stratification and management of LGG.

## Introduction

The Duchenne muscular dystrophy gene (*DMD*) is located within a common fragile site on the X chromosome and at > 2 Mb in size is one of the largest known human genes^1,2^. The major protein product of the gene is the 427 kDa dystrophin protein whose primary function is to maintain skeletal muscle integrity by connecting the actin cytoskeleton to the extracellular matrix^3^. The resulting dystrophin-associated protein complex (DAPC) is essential for muscle function. Smaller dystrophin proteins (Dp) are produced from the *DMD* gene through independent promoter usage and are named according to their size in kDa^3^. Dp71 is a ubiquitous dystrophin protein with prominent neuronal and glial expression throughout the cortex and cerebellum at all stages of development^4^. Dp71 is alternatively spliced to generate four major isoforms which have a large functional diversity including neuronal differentiation, adhesion, cell division, excitatory synapse organisation, nuclear scaffolding and DNA repair^4,5^.

Mutations in the *DMD* gene can cause Duchenne or Becker muscular dystrophy depending on whether the reading frame, and therefore dystrophin protein expression, is disrupted^3^. Besides this major role in muscular dystrophy, a growing body of evidence suggests that *DMD* gene mutations and/or changes in *DMD* gene expression are associated with the development, progression and survival outcomes of both myogenic and non-myogenic cancers including tumours of the central nervous system^6–9^. In a study by Luce et al*. DMD* ranked within the top 10% of differentially expressed genes in several tumour versus normal tissue comparisons. In the nervous system, *DMD* was significantly overexpressed in ependymomas and astrocytomas, but was significantly under-expressed in medulloblastoma and non-significantly under-expressed in glioblastoma^8^. In our own preliminary study to determine the effect of high versus low *DMD* gene expression on survival outcomes across all cancers, we found that low-grade glioma (LGG) returned one of the highest hazard ratios. In agreement with Luce et al*.* we posited that these findings may be attributed to Dp71 given it is the predominant *DMD* gene product in the brain and has known roles in proliferation, invasion and migration^5,7,10^. We therefore aimed to undertake the first dedicated study of the involvement of *DMD* and its individual gene products in LGG.

According to the revised 2016 WHO criteria for the classification of central nervous system tumours^11^, LGG comprise grade I and II astrocytomas and oligodendrogliomas. A not otherwise specified (NOS) category is also permissible for e.g. a prior diagnosis of oligoastrocytoma which is no longer considered a distinct subtype. Grade III and IV gliomas are considered high-grade, the latter includes the most aggressive type known as glioblastoma, or glioblastoma multiforme (GBM). LGGs account for approximately 11% of all primary brain tumours with a peak incidence between 35 and 44 years of age and an average mean survival time of 7 years^11,12^. In most cases LGGs progress to fatal high-grade tumours within 5–10 years^13^. Treatment and management is difficult due to uncertainty over progression and lack of best practice^13^. There is an urgent unmet scientific and clinical need to characterise the mechanisms responsible for LGG development and progression. Genotypic features and prognostic markers such as isocitrate dehydrogenase (IDH) mutation status as well as the presence/absence of the chromosomal 1p/19q co-deletion are used to aid the diagnosis and management of LGG^11^. Both IDH mutation and 1p/19q co-deletion are predictive of better survival but the detailed mechanisms are unknown^14^. Recent changes in surgical practice have improved survival rates with early resections now considered part of the overall standard of care for LGG^15,16^. However, recurrence remains common and is unpredictable with significant variability across tumour subtypes which presents a continued dilemma for treatment and management^17^.

We present, for the first time, an in-depth bioinformatic analysis of the association of *DMD* gene expression with overall survival in LGG. We show that *DMD* is an independent prognostic marker for LGG where high expression is linked to poor overall survival in IDH mutant LGG. Furthermore, we demonstrate that the expression of multiple *DMD* gene products, including Dp71, are also associated with LGG survival outcomes and have differential biological effects relevant to the pathogenesis of LGG.

## Results

### High *DMD* gene expression is associated with poor survival in low-grade glioma and is an independent prognostic marker

**Figure 1:**
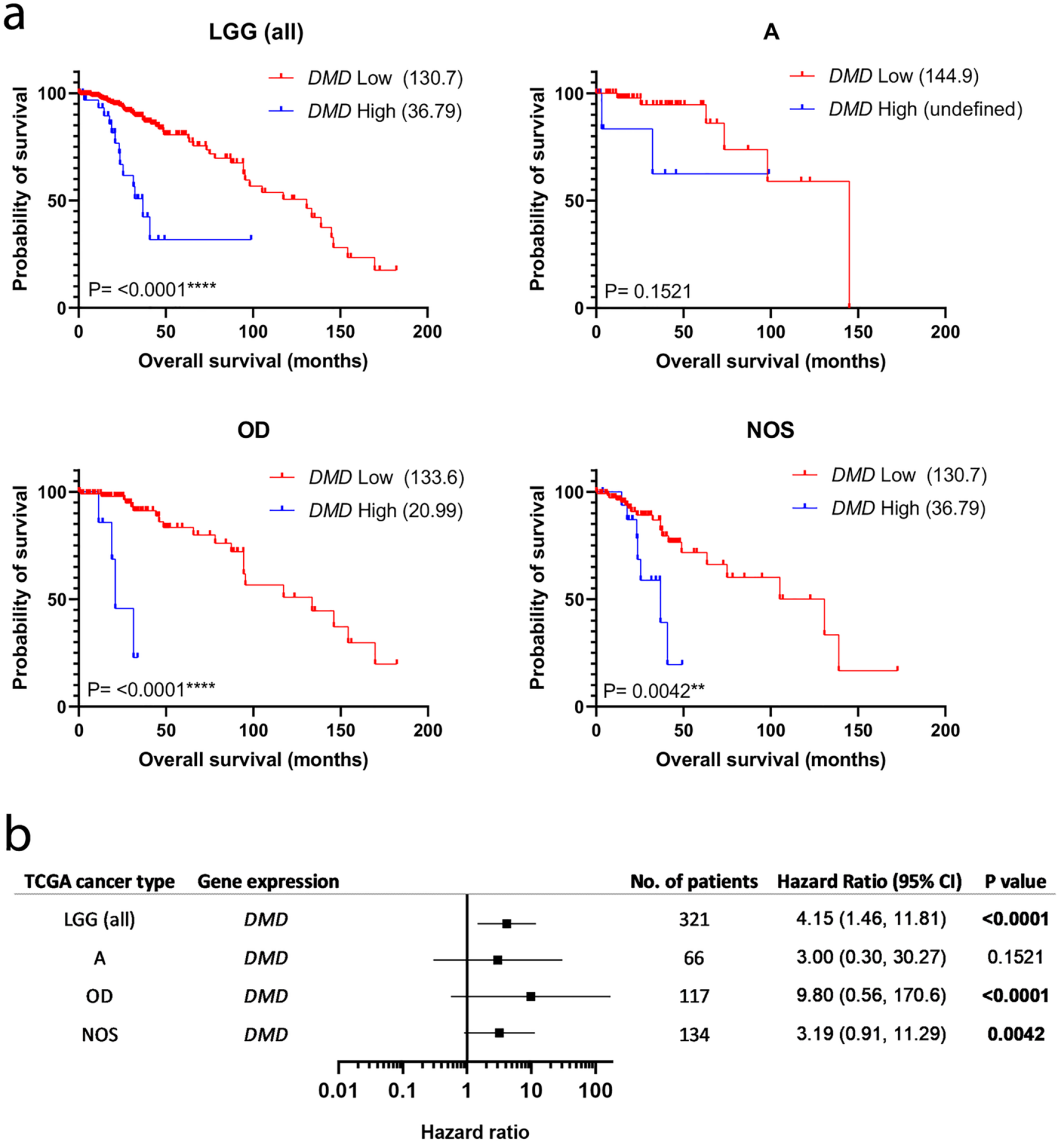
High *DMD* expression is significantly associated with poor survival in LGG. (**a**) TCGA RNAseq data from WHO grade II LGG cases was dichotomised into high (blue) and low (red) *DMD* expressing groups and survival analysis performed in GraphPad using the log-rank test. Tumour subtype analysis was also performed. Numbers in brackets are median overall survival times in months. (**b**) Forest plot revealing the log-rank hazard ratio with 95% confidence intervals and number of patients for each group. *A* astrocytoma, *OD* oligodendroglioma, *NOS* not otherwise specified.

To explore the association of *DMD* gene expression in LGG, we analysed RNAseq data from a LGG (WHO grade II) TCGA dataset using an independent bioinformatic pipeline and cut-point approach (Fig. 1). We confirm in univariate analyses that high *DMD* is associated with poor survival in LGG (HR 4.15; 95% CI 1.46, 11.81; P = < 0.0001). The median overall survival for the high *DMD* group was 36.79 months compared to 130.7 months for the low *DMD* group, a difference of over 7 years. We performed a tumour subtype analysis to determine whether the survival associations are limited to or differ between subtypes (Fig. 1). Univariate subtype analysis revealed that high *DMD* is associated with poor survival across all subtypes which was significant for all except astrocytoma (A) which constituted the smallest number in the cohort (n = 66). Oligodendroglioma had the highest increased risk of poor survival, though with a wide confidence interval (HR 9.80; 95% CI 0.56, 170.6; P = < 0.0001). Multivariate analysis including tumour subtype as a factor revealed that *DMD* expression remained significant whilst tumour subtype did not (P = 0.005, Supplementary Table S1). The effect of *DMD* gene expression on glioma survival was specific to only WHO grade II LGG; high-grade (grade III) anaplastic tumours and high-grade (grade IV) glioblastoma TCGA datasets did not result in any significant survival differences when stratified by high/low *DMD* expression (Supplementary Fig. S1). To validate these findings, RNAseq data from the Chinese Glioma Genome Atlas (CGGA) was taken as an independent dataset and replication cohort (Supplementary Fig. S2). Whilst *DMD* expression is associated with survival of low-grade II LGG in the TCGA dataset, the CGGA dataset replicated this finding for only for high-grade III LGG. Overall however, the CGGA data does confirm that *DMD* expression is not significantly associated with survival in the most invasive and aggressive grade IV glioblastoma.

We next performed a multivariate analysis to determine whether *DMD* expression remains significantly associated with survival in a model containing significant clinicopathological variables. In the cohort we used, age at diagnosis and IDH mutation status both had independent Kaplan–Meier statistics of P = < 0.01 and were significantly associated with survival in agreement with existing literature^14,18,19^ (Supplementary Fig. S3). Note the TCGA dataset splits IDH mutated patients into 1p/19q co-deleted and non-co-deleted groups since this chromosomal co-deletion is also known to have a strong effect on LGG survival outcomes^14,20^. *DMD* expression remained significant in a multivariate analysis with age at diagnosis (< 41 years; > 41 years) and IDH mutation/co-deletion status (wild-type; IDH mutant co-deleted and IDH mutant non-co-deleted) and is therefore independently prognostic of these for LGG (Supplementary Fig. S3, P = 0.02; HR 2.12; 95% CI 1.13, 3.99). IDH mutation status also remained significant in this model but age at diagnosis did not. This provides strong confirmation of a potential role for *DMD* in LGG tumourigenesis and/or disease progression that warrants further investigation.

### *DMD* gene expression further stratifies IDH mutant LGG

**Figure 2:**
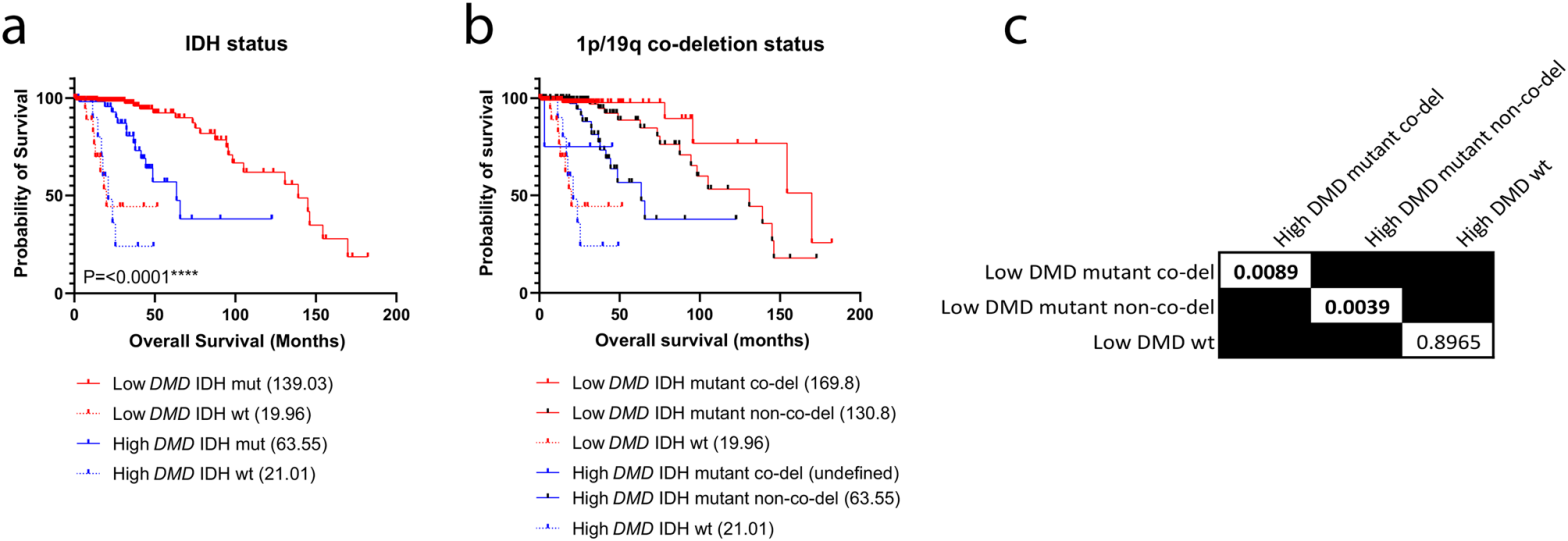
Pairwise comparison of Kaplan–Meier curves identifies a subgroup of IDH mutated LGG patients with poor survival. The Kaplan–Meier survival curves for high (blue) vs low (red) *DMD* expression for (**a**) IDH mutation status and (**b**) 1p/19q co-deletion status were compared. Numbers in brackets are median overall survival times in months. The table provides the log-rank test P values for each planned comparison from (**b**); the alpha value was adjusted to 0.017 to correct for multiple testing.

Since both IDH mutation status and 1p/19q co-deletion are used clinically to aid prognosis we also investigated, using pairwise comparisons of Kaplan–Meier curves, the survival outcomes of high vs low *DMD* expression in all IDH and co-deletion status groups (Fig. 2). Low *DMD* expression offers a particularly significant survival advantage for IDH mutated patients regardless of 1p/19q co-deletion status compared with high *DMD* (Fig. 2a). In non-1p/19q co-deleted patients, the difference in median survival between high and low *DMD* groups was 67.25 months (> 5 years, P = 0.0039, Fig. 2b,c). The median survival was undefined for the high *DMD* co-deleted group which contained only four patients, but the same trend is still observed between high and low *DMD* for 1p/19q co-deleted patients (P = 0.0089). *DMD* expression was not significantly associated with survival in IDH wild-type patients with both *DMD* high and *DMD* low groups having an overall median survival of approximately 20 months (P = 0.8965); this was also replicated in the CGGA dataset (Supplementary Fig. S4). Overall this data demonstrates that *DMD* expression further stratifies IDH mutant patients (P = 0.0039 for non-1p/19q co-deleted patients and P = 0.0089 for co-deleted patients). Interestingly, high *DMD* expression appears very rare in co-deleted patients (4% [4/107] co-deleted vs 34% [55/161] non-co-deleted), this was also replicated in the CGGA cohort where only 3% (2/58) of co-deleted patients had high *DMD* expression compared to 31% (31/101) of non-co-deleted patients.

**Figure 3:**
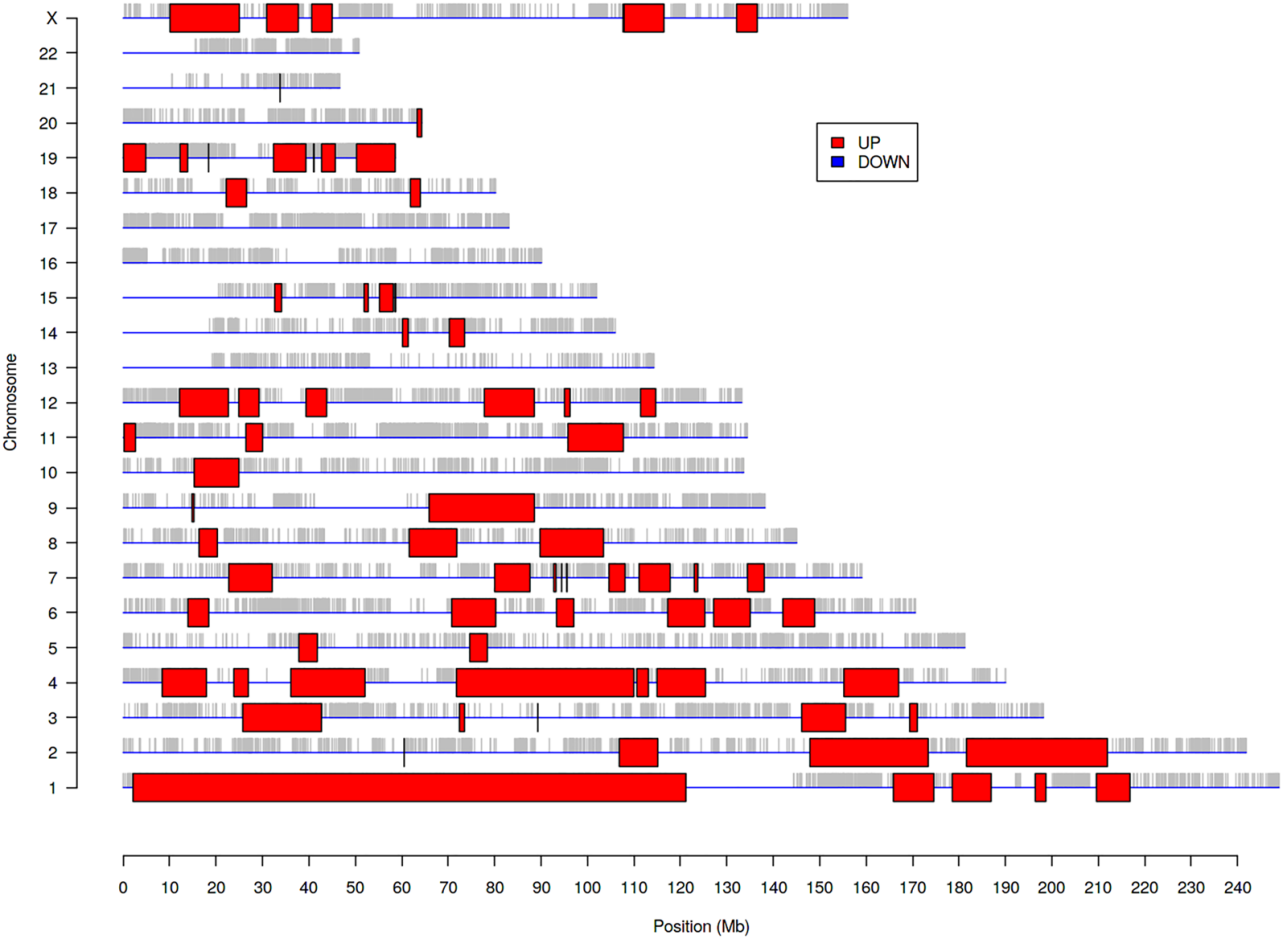
Genomic regions significantly dysregulated in WHO grade II LGG cases with high vs low *DMD* expression. Significantly altered regions are highlighted with coloured boxes.

Given the above findings and the established link between 1p/19q co-deletion and LGG survival, we detected the differentially expressed genomic regions in high versus low *DMD* samples using Position RElated Data Analysis (PREDA^21^). Many statistically significant chromosomal regions with expression changes among neighbouring genes were identified (Fig. 3). Notably, genes covering the whole of the p-arm of chromosome 1 are up-regulated as well as many on chromosome 19q. Thus, 1p/19q genes are upregulated in patients with high *DMD* expression which worsens survival outcomes for these patients. High *DMD* expression almost halved the overall median survival of non-1p/19q co-deleted patients we observed in Fig. S3a from 105 to 64 months (> 3-year difference) whilst low *DMD* expression maintained the more favourable median survival time (131 months) expected of this group.

### Network and pathway analysis of differentially expressed genes (DEGs) implicates development and ribosome biogenesis

**Figure 4:**
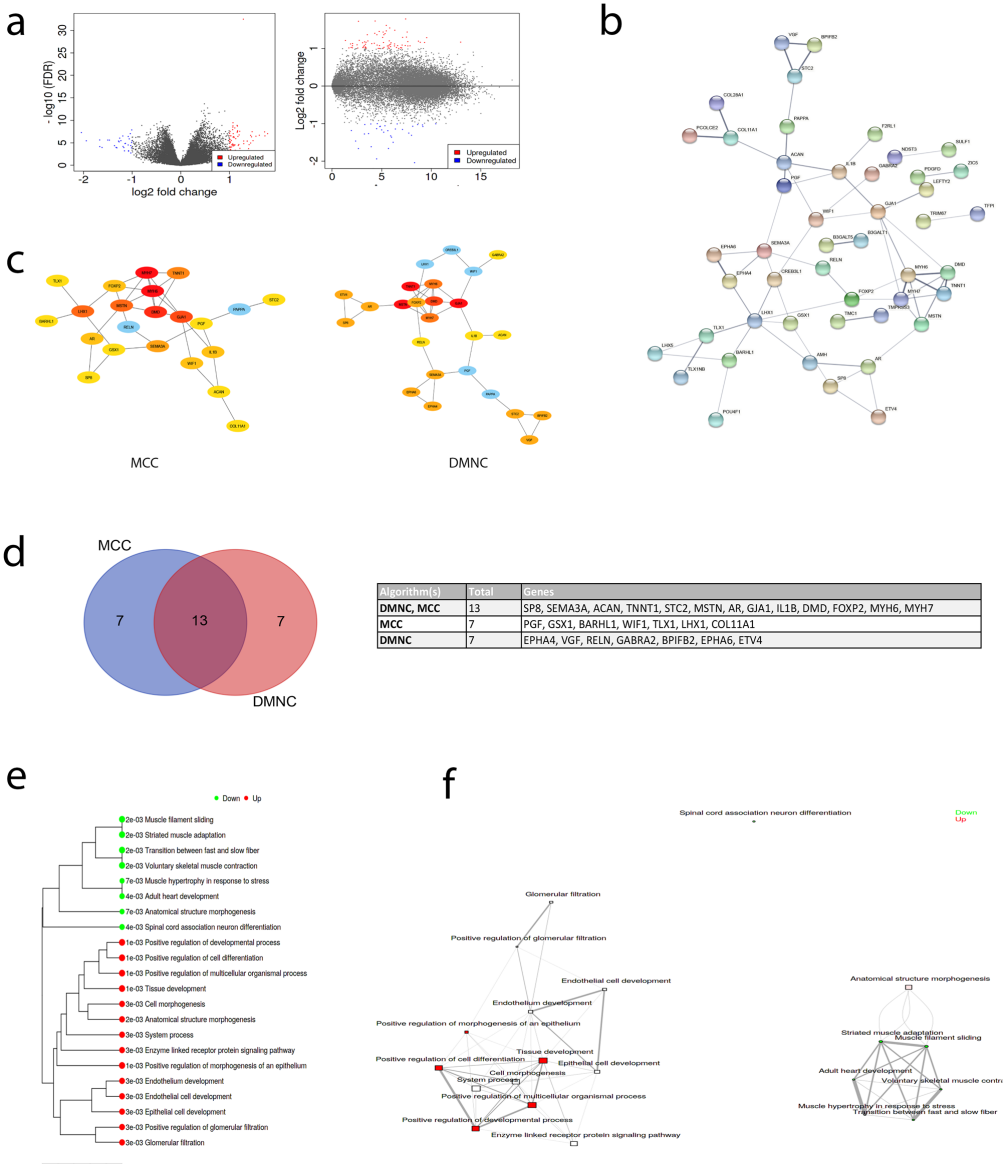
Exploratory analysis of the DEGs in LGG cases with high versus low *DMD* expression. (**a**) Volcano and M-A plots of the log fold change of all genes. Upregulated and downregulated genes are indicated by red and blue points respectively. (**b**) STRING PPI network of DEGs with 60 edges (versus 20 expected) and an enrichment P value of 7.92e−13. Edge thickness indicates confidence, disconnected nodes are hidden. (**c**) Identification of significant hub genes from DEGs using the Cytohubba MCC and DMNC algorithms within Cytoscape. Colour represents ranking based on corrected P values from red to yellow, the expanded subnetwork is displayed by blue nodes. (**d**) Venn analysis of both sets of hub genes reveals 13 common significant hub genes. (**e**) Network tree visualisation of the enriched pathways in DEGs using the GO biological processes annotation, dot size corresponds to adjusted P values. (**f**) Visualisation of the relationship among enriched GO categories. Connected gene sets share more genes, colour of node represents adjusted P values.

To aid future investigation into the functional role(s) of the *DMD* gene in LGG tumourigenesis we undertook a detailed bioinformatic analysis of the DEGs in the TCGA LGG (grade II) cases with high versus low *DMD* expression. We used an integrated web application, iDEP, for data pre-processing and to identify the DEGs (DESeq2 method). The volcano and M-A plots show a subtle transcriptomic response (Fig. 4a). A total of 106 DEGs were identified. These included 32 down-regulated genes and 74 up-regulated genes (Fig. 4a, Supplementary Data S1). The Search Tool for the Retrieval of Interacting Genes (STRING) was used to identify the protein–protein interaction (PPI) network connectivity of the 106 DEGs (Fig. 4b). The resulting PPI network contained 101 nodes forming a core of 47 highly connected proteins with 60 edges, the PPI enrichment P-value was < 0.0001 (7.92e−13) using a median confidence score of 0.4. The expected number of edges for a random set of proteins of similar size was 20 which strongly suggests functional intersection of the identified DEGs. STRING additionally detected that the homeobox domain is overrepresented in the proteins encoded by the down-regulated genes hinting at a role for these DEGs in embryonic development. To identify hub genes within the DEGs, we used the Cytohubba plugin on Cytoscape. The top 20 nodes were ranked by the Maximal Clique Centrality (MCC) and Density of Maximum Neighbourhood Component (DMNC) algorithms (Fig. 4c). The list of hub genes from each algorithm are displayed (Fig. 4d) where 13 genes were common to both algorithms, several of which do indeed have roles in development.

**Figure 5:**
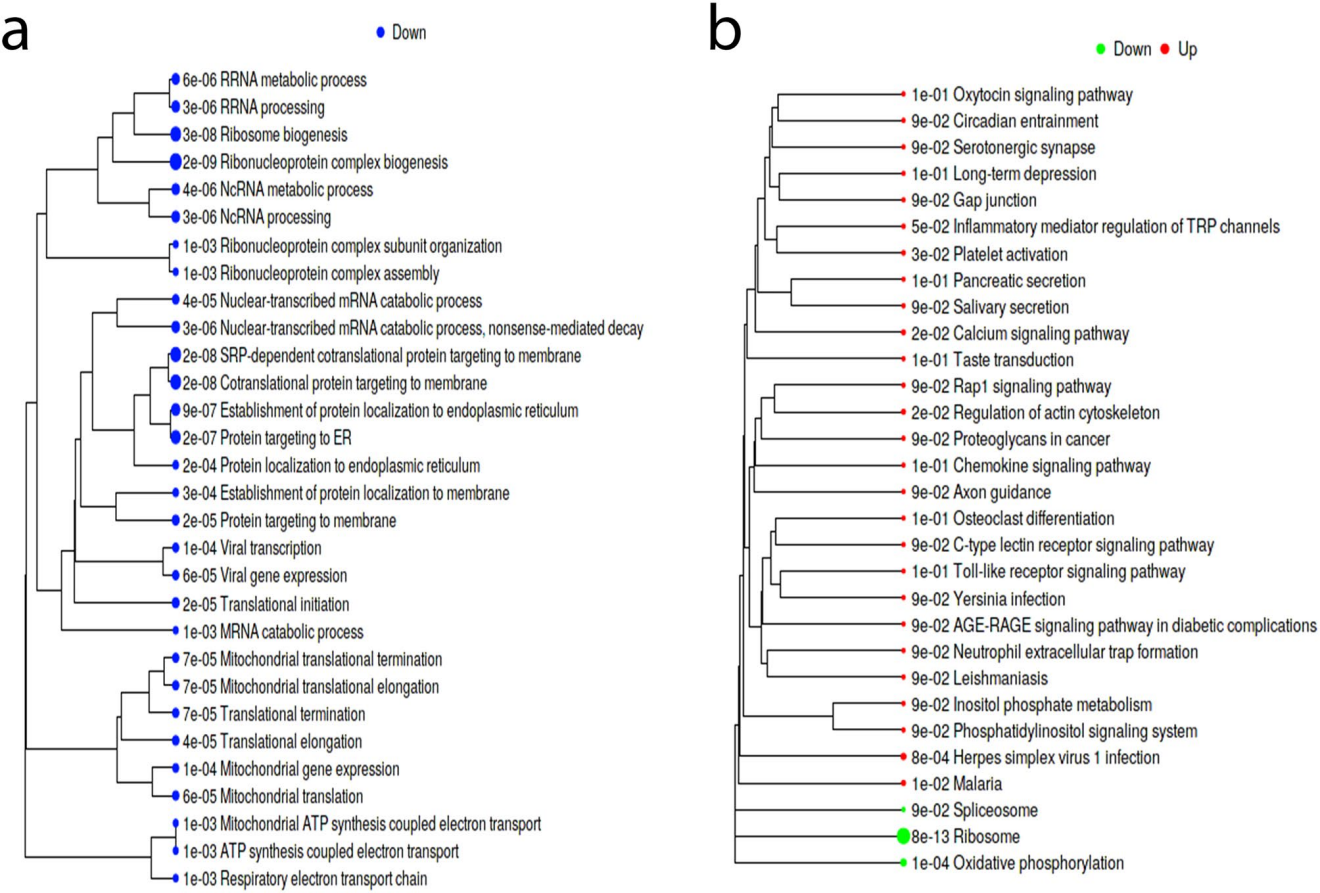
Pathway analysis reveals high *DMD* expression downregulates biological processes related to ribosome biogenesis. iDEP was used to conduct pathway analysis using the generally applicable gene set enrichment (GAGE) method and the genes were annotated according to GO biological processes (**a**) or with Kyoto Encyclopaedia of Genes and Genomes (KEGG, **b**)^31^. The size of the dot corresponds to the adjusted P values.

To examine the functional annotation of the DEGs we used the enrichment analysis (gene ontology [GO] biological processes) for DEGs tool in iDEP (Fig. 4e,f). The results are in line with the functions of the identified hub genes in Cytoscape. The up-regulated genes are enriched in processes relating to development and morphogenesis; the down-regulated genes are also enriched in aspects of development including neurodevelopment and processes relating to muscle contraction. As well as analysing the function of the DEGs, we performed a separate pathway analysis using the fold-change values of all the genes in our dataset to identify coherently altered pathways upon high vs low *DMD* expression. We used the generally applicable gene set enrichment (GAGE) method within iDEP and the genes were annotated either according to GO biological processes or with Kyoto Encyclopaedia of Genes and Genomes (KEGG) (Fig. 5). The most significant result returned by the KEGG annotation was a down-regulation of the ribosome pathway which is reflected also in the GO biological processes annotation which returned high significance for terms such as ribosome biogenesis. Thus, in high vs low *DMD* cases, ribosome pathways are downregulated and selected DEGs are enriched in processes relating to development.

**Figure 6:**
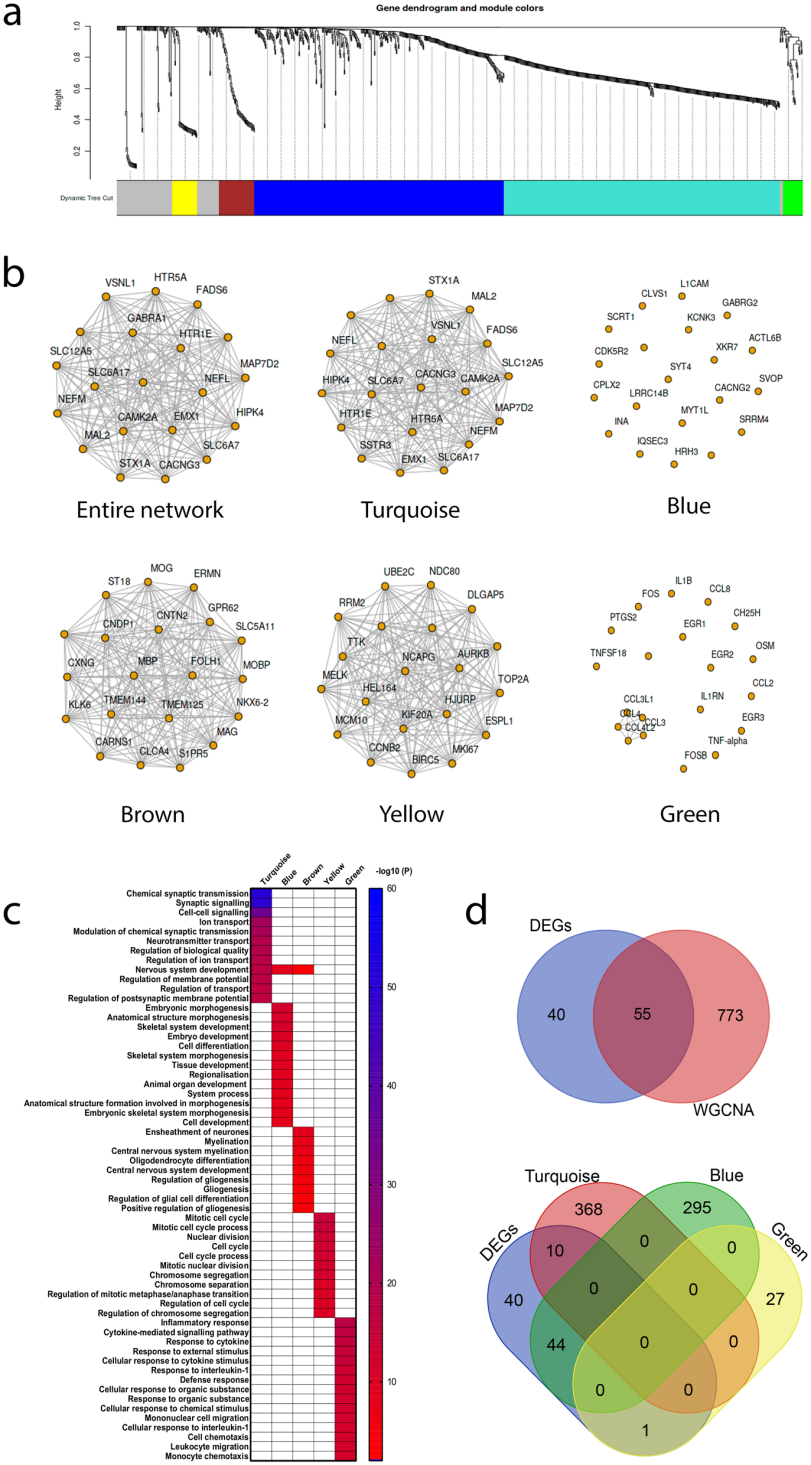
WGCNA of the TCGA WHO grade II LGG dataset identified a network of 882 genes divided into five co-expression modules. (**a**) Gene dendrogram. Colours are randomly assigned except grey which represents areas unassigned to a module. (**b**) Networks of the top 20 genes for the entire network and each module individually. (**c**) Visualisation of the GO enrichment analysis for each module; heatmap was produced in GraphPad. (**d**) Venn analysis to identify common genes returned by both WGCNA and DEG analysis.

We additionally assessed the co-expression behaviour of the top 1000 variable genes across all samples using weighted gene co-expression network analysis (WGCNA, Fig. 6). This analysis identifies groups of genes whose expression levels are similar and tend to co-activate. A consensus LGG network of 882 genes that are correlated in all samples was divided into five modules (Supplementary Data S2) and the network of the top 20 genes displayed for each module (Fig. 6a,b). GO enrichment analyses was performed on the five modules (Fig. 6c). The 402 turquoise module genes are significantly involved in biological processes related to cell signalling, synaptic signalling and transmission and nervous system development. The 364 blue module genes are involved in many aspects of embryonic, tissue and cell morphogenesis and development. The 52 brown module genes are involved in myelination, gliogenesis and glial cell differentiation. The 36 yellow module genes are involved in mitosis, nuclear division and regulation of the cell cycle. Finally, the 28 green module genes are involved in inflammatory response, chemotaxis and response to cytokine stimulus. It is to be noted that the large turquoise and blue modules are less specific in nature with the blue module having a lower degree of co-expression. The DEGs identified above were well represented with 55 DEGs contained within the entire network (Supplementary Table S2). Venn analysis reveals that 44 of the DEGs are found in the blue module linked to morphogenesis and development; the remaining DEGs are found in the turquoise (10 DEGs) and green (1 DEG) modules (Fig. 6d). Our WGCNA analysis confirms a role for the DEGs identified above in development and in the same biological processes as the commonly co-expressed genes in LGG. WGCNA additionally highlights relevant and specific genes involved in gliogenesis and glial cell differentiation (brown module) and cell growth and division (yellow module). In summary, high *DMD* expression is significantly linked to poor survival outcomes in IDH mutant LGG. In patients with high *DMD* expression, ribosome pathways are downregulated and selected DEGs are enriched in processes relating to morphogenesis and development.

### The association of *DMD* gene expression with LGG survival is replicated by multiple *DMD* gene products

**Figure 7:**
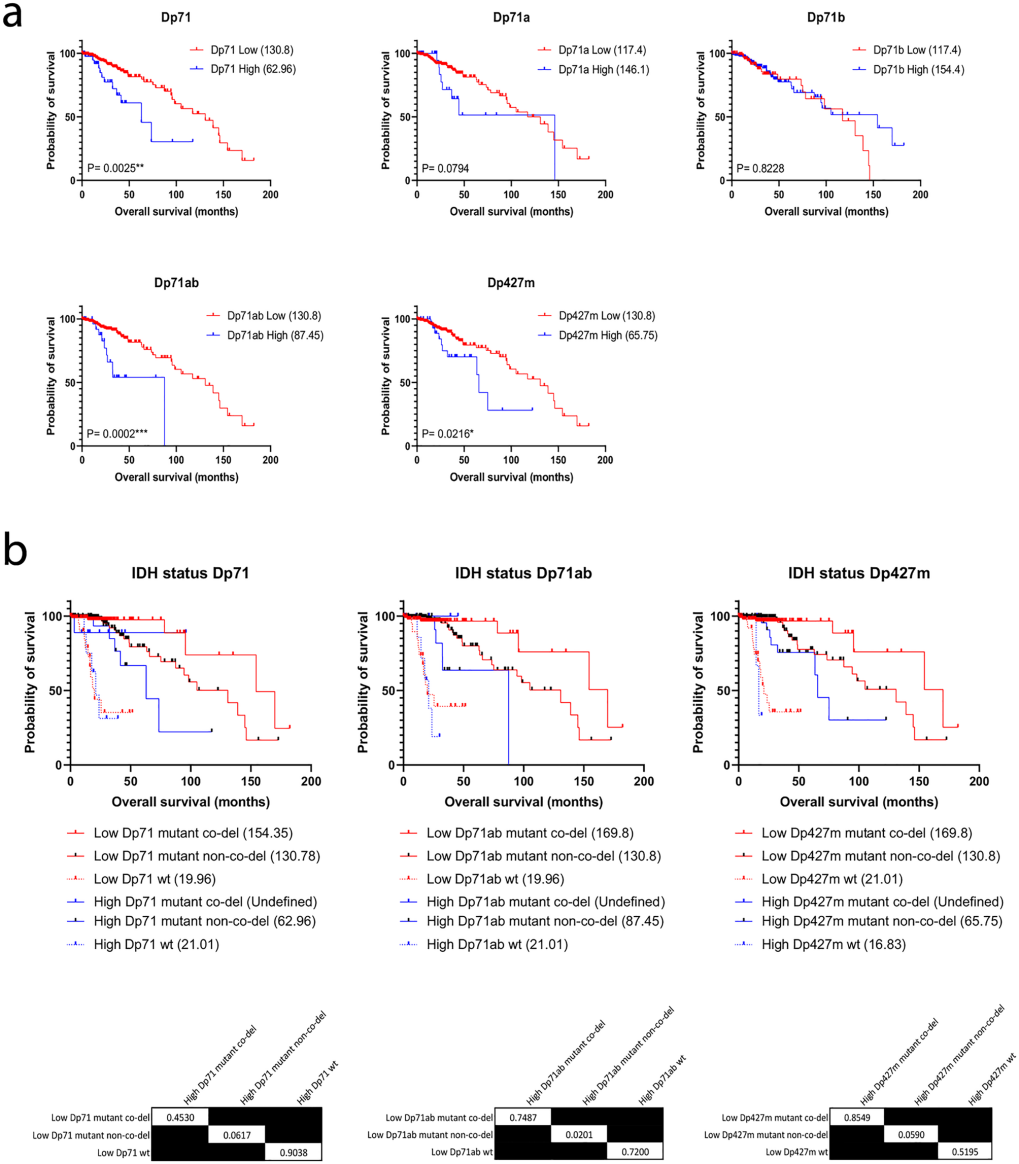
The expression of multiple *DMD* gene products are significantly associated with LGG survival outcomes. (**a**) LGG TCGA RNAseq data for each *DMD* isoform was dichotomised into high (blue) and low (red) expression groups and survival analysis performed in GraphPad using the log-rank test. Numbers in brackets are median overall survival times in months. (**b**) Kaplan–Meier survival curves for high (blue) vs low (red) Dp71, Dp71ab or Dp427m expression for each IDH mutation status group were compared. Numbers in brackets are median overall survival times in months. The tables provide the log-rank test P values for each planned comparison; the alpha value was adjusted to 0.017 to correct for multiple testing.

The *DMD* gene is complex. As well as the major full-length 427 kDa dystrophin essential for muscle function, several other *DMD* gene variants are produced from independent promoters, many of which are themselves alternatively spliced to produce multiple isoforms^3^. Although literature has previously linked the *DMD* gene to numerous cancers^6^, none have considered the many gene products produced by the *DMD* gene and the extent of their contributions to pathogenesis and associated survival outcomes. To begin to identify the relative contribution of individual *DMD* gene products we extracted RNAseq isoform data from the same TCGA cases analysed above. We plotted individual *DMD* gene product expression levels for all patients and determined that the four major Dp71 isoforms (Dp71, Dp71a, Dp71b and Dp71ab) and Dp427m are the most abundant in LGG tissue (Supplementary Fig. S5). There is a weak positive correlation between their expression and total *DMD* expression; this was significant for Dp71a, Dp71ab and Dp427m (Supplementary Fig. S5). We therefore sought to determine the expression of which Dp71 isoform(s) and/or Dp427m gene products were associated with LGG survival. To test this, we repeated our analysis above using individual *DMD* gene product expression data (Fig. 7a). We found that high expression of the Dp71, Dp71ab and Dp427m gene products were significantly associated with poor LGG survival. These findings were replicated across the oligodendroglioma subtype accounting for multiple testing (Supplementary Fig. S6). We moved forward with a detailed analysis of Dp71, Dp71ab and Dp427m since they were significant across the whole LGG cohort.

In a multivariate analysis with the significant clinicopathological variables of age at diagnosis (< 41 years; > 41 years) and IDH mutation status (wild-type; IDH mutant co-deletion and IDH mutant non-co-deletion), none of the individual gene products remained significant (Supplementary Tables S3–S6). However, pairwise comparisons of the Kaplan–Meier curves for all IDH and co-deletion status groups show that in keeping with our findings for *DMD* expression above, low Dp71, Dp71ab and Dp427m expression offers a survival advantage for IDH mutated patients compared with high expression (Fig. 7b). As with *DMD,* the expression of Dp71, Dp71ab and Dp427m were clearly not associated with survival in IDH wild-type patients with each *DMD* low and *DMD* high group having an overall median survival time of approximately 20 months (e.g. P = 0.9038 for Dp71). Thus, high Dp71, Dp71ab and Dp427m are significantly associated with poor survival in IDH mutant LGG but do not offer any additional prognostic utility beyond that of total *DMD* expression.

### High expression of individual *DMD* gene products have differential biological effects relevant to the pathogenesis of LGG

**Figure 8:**
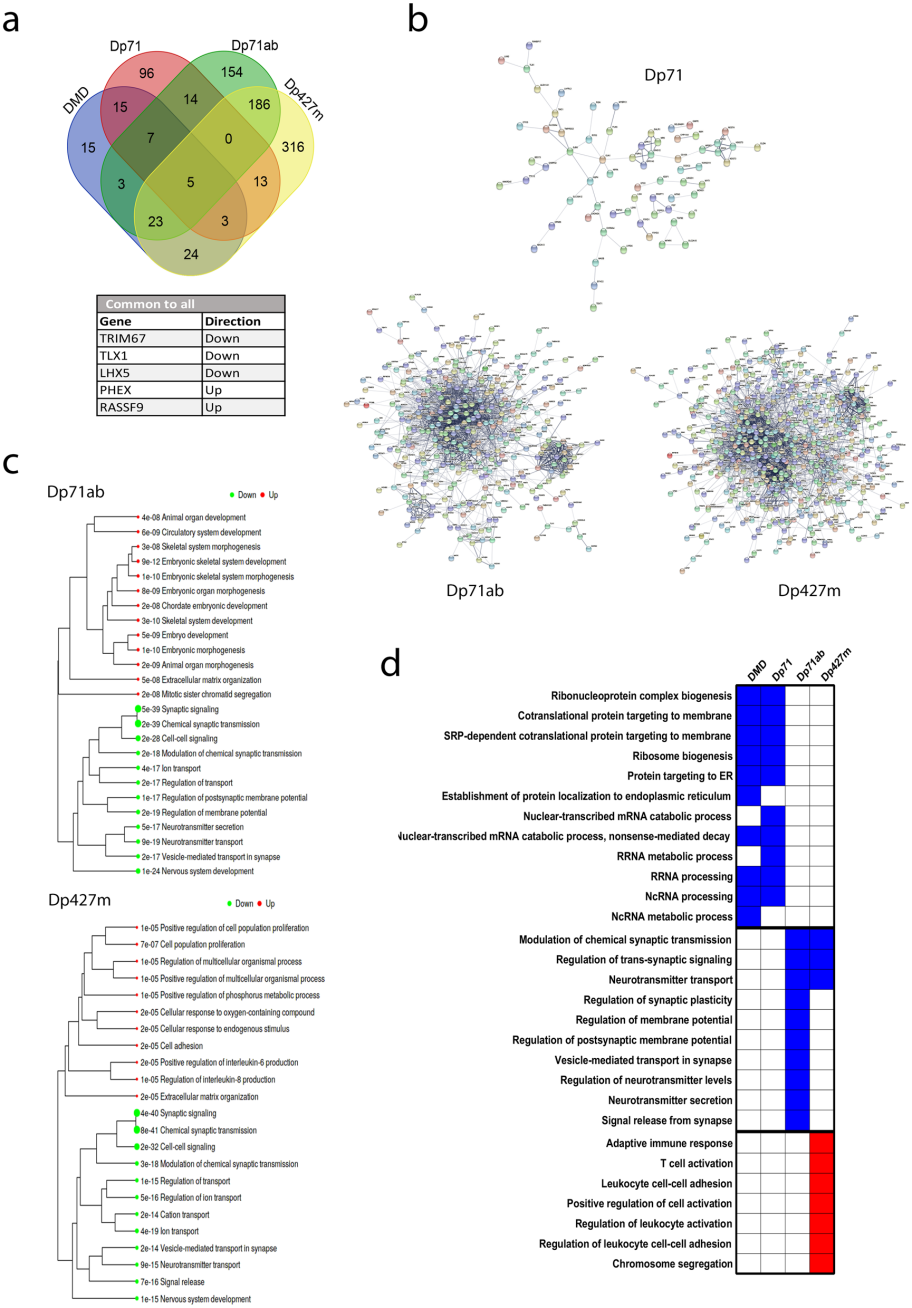
Exploratory analysis of the DEGs in LGG cases with high versus low Dp71, Dp71ab and Dp427m expression. (**a**) Venn analysis of the DEGs identified from high vs low overall *DMD*, Dp71, Dp71ab and Dp427m expression. The five DEGs common to all are listed. (**b**) STRING PPI networks of DEGs. Edge thickness indicates confidence, disconnected nodes are hidden. (**c**) Network tree visualisation of the enriched pathways in DEGs using the GO biological processes gene annotation, dot size corresponds to adjusted P values. Note no significant enrichment was found for Dp71 DEGs. (**d**) Pathway analysis using GO biological processes gene annotation. The heatmap was produced in GraphPad and displays the top 10 GO terms for high vs low *DMD*, Dp71, Dp71ab and Dp427m. Upregulated (red) and downregulated (blue) pathways are indicated.

We repeated DEG and pathway analysis on the TCGA RNAseq dataset configured for either high vs low Dp71, Dp71ab or Dp427m expression (Fig. 8). The number of DEGs identified for high vs low Dp71 was 167; 30 of which are in common with those identified for high vs low *DMD* expression (Fig. 8a). A more extensive transcriptomic response was observed with high Dp71ab and Dp427m expression where 422 and 617 DEGs were identified with 38 and 55 in common to *DMD*, respectively. The full list of DEGs are provided (Supplementary Data S1) and the PPI networks for each set of DEGs (Fig. 8b) illustrate very large and extensively connected networks for Dp71ab (380 nodes, 2205 edges [497 expected], PPI enrichment value P = < 1.0e−16) and Dp427m (549 nodes, 2856 edges [1058 expected], PPI enrichment value: P = < 1.0e−16) in comparison to Dp71 (147 nodes, 78 edges [35 expected], PPI enrichment value P = 3.84e−10). The hub genes identified for each network are listed in Supplementary Table S7 and the pathways enriched in DEGs in Fig. 8c, note no significant enrichment was found for the Dp71 DEGs. There is similarity between Dp427m and Dp71ab DEGs (214 in common), with both sets of DEGs significantly enriched in synaptic signalling and nervous system development pathways. These pathways were also significantly altered between high vs low *DMD* expression groups and were also returned in the WGCNA of the whole LGG dataset.

Pathway analysis of all the genes, independent of DEGs, identifies three pathway clusters differentially altered depending on which *DMD* gene product is highly expressed (Fig. 8d). High *DMD* and high Dp71 expression significantly down-regulates ribosome biogenesis and protein targeting pathways whilst high Dp71ab and Dp427m expression significantly down-regulates pathways related to synaptic signalling. High Dp427m expression additionally up-regulates immune and leukocyte cell pathways. Some of these pathways were also featured in the WGCNA modules (Fig. 6).

PREDA on the high vs low Dp71 and Dp427m datasets resulted in a similar level of expression changes across different chromosomal regions to that observed with high vs low *DMD*, including a significant up-regulation of genes across a large region of chromosome 1p (Supplementary Fig. S7). However, when we conducted PREDA on the high vs low Dp71ab dataset, no significant regions were found. In summary, our data indicates that the expression of multiple *DMD* gene products are linked to overall survival in LGG which have differential biological effects relevant to the pathogenesis of LGG.

### Dystrophin is expressed in the cytoplasm and nucleus of glial cells in LGG

**Figure 9:**
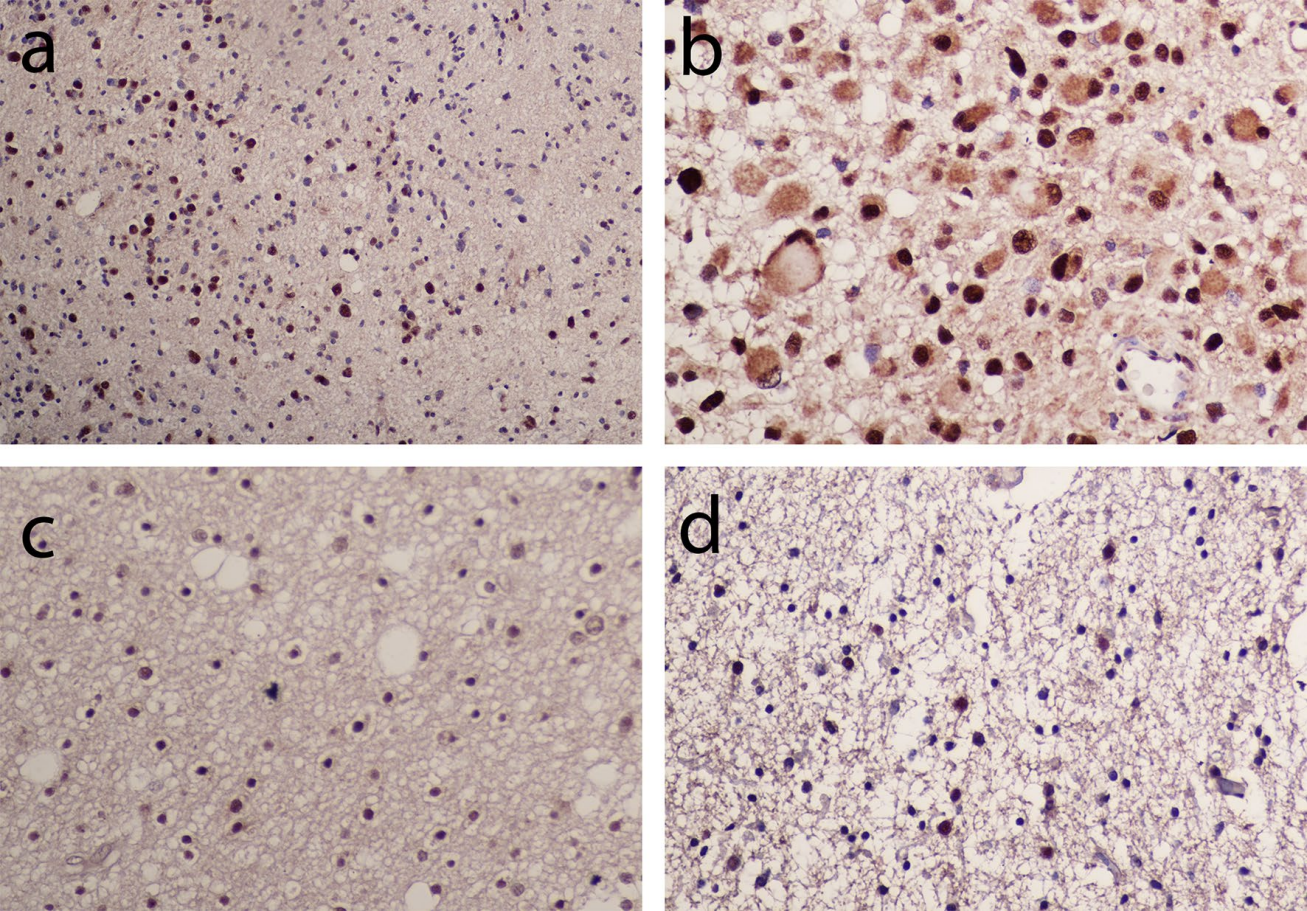
Immunohistochemistry for dystrophin in LGG. Representative images of grade II astrocytoma (**a**), gemistocytic astrocytoma (**b**), oligodendroglioma (**c**) and NOS (**d**). Images were taken at × 20 magnification except for image (**b**) which was taken at × 40 magnification to better visualise the gemistocytic staining.

To confirm whether dystrophin protein is expressed in LGG tissue, and in which cell types, we conducted a pilot immunohistochemistry study on a cohort of 24 LGG cases (18 astrocytoma, one oligodendroglioma and five NOS) using a C-terminal dystrophin antibody which detects all dystrophin proteins. Nine out of the 24 cases were IDH mutant and 15 were IDH wild-type. We observed dystrophin expression in both the cytoplasm and nucleus of glial cells within LGG (Fig. 9). Overall dystrophin staining was most predominant in the nucleus. Notably, the number of positively stained cells, as well as the intensity of nuclear, cytoplasmic and neuropil staining, was variable among cases but the cohort is too small to provide a meaningful survival analysis. These results confirm the feasibility of assessing the association of dystrophin protein expression with survival in future studies of large LGG cohorts.

## Discussion

Our results identify a new subset of IDH mutant LGG patients who have a significantly poor survival outcome. IDH mutant LGG patients with high *DMD* expression in their tumours survive approximately 6 years less than those with low expression; this appears to be particularly relevant for non-1p/19q co-deleted patients since high *DMD* expression was exceptionally rare amongst 1p/19q co-deleted cases. In non-co-deleted patients, survival outcomes are already poorer than for co-deleted patients^20^ and we demonstrate that if high *DMD* expression occurs then the outcome is even worse. Interestingly, we found that 1p/19q genomic regions are co-ordinately up-regulated in LGG cases with high *DMD* expression which may contribute to pathogenesis. Whilst we have confirmed dystrophin protein is expressed in LGG tumour tissue, further work is needed to experimentally confirm our findings in for e.g. a large immunohistochemical cohort as well as to investigate the potential involvement of *DMD* gene product(s) in LGG tumourigenesis. It is unknown for example whether the *DMD* gene plays a driver role in the cancers it has been linked to, or whether what we (and others^6^) have described are passenger effects. Determining the prognostic utility of *DMD* gene expression and the drug target potential of specific *DMD* gene products may lead to improved risk stratification and the development of new therapeutic strategies for LGG. The Dp71 isoforms have not been studied in low grade glioma before and studies on *DMD* in cancer have not fully considered the complexity of the gene and the roles its individual gene products may play.

Of note, in our CGGA replication cohort, the above findings were replicated in a different grade of tumour (high-grade III versus low-grade II). The decision by neuropathologists as to whether to score a tumour grade II or grade III is difficult and very subjective; practices may vary world-wide. Whilst both cohorts are well matched in terms of age of onset, sex and IDH mutation status, there may also be additional population-specific differences and/or mutations that might be relevant for stratifying the patients before considering *DMD* as a marker for prognosis. We think it of importance however, that in our study both cohorts confirmed that *DMD* expression is not significantly associated with survival in the most invasive and aggressive grade IV glioblastoma.

Our findings extend those of Luce et al*.* who reported in 2017 that *DMD* is significantly overexpressed in astrocytoma and non-significantly underexpressed in glioblastoma^8^. One study has linked Dp71 to high-grade glioblastoma where Dp71 expression appears decreased in glioblastoma cell lines and inversely correlated with the Ki-67 tumour proliferative index in tumour tissue^22^. However, the Ki-67 index is considered a poor predictor of survival in glioblastoma^23^ and further work is needed to determine the true effect (if any) of altered Dp71 expression on survival outcomes in high-grade glioblastoma. Our data from two independent cohorts suggests that overall *DMD* expression is significantly associated with survival outcomes in only the less invasive, IDH mutant, gliomas indicating a likely role for *DMD* in the early stages of gliomagenesis.

Both Dp427m and the Dp71 isoforms form DAPCs in the brain^4^. Other components of this complex such as the dystroglycans have also been linked to the progression of primary brain tumours including gliomas^24,25^. Day et al*.* demonstrated that dystroglycan plays a role in maintaining tumour supporting glioma stem cells in the extracellular matrix (ECM)-rich perivascular niche. Surface markers on these glioma stem cells are strong independent prognostic markers of low-grade glioma progression and survival^26^. Thus, considering our own data implicating ECM-linked dystrophin proteins in low-grade glioma, an area of future investigation could be whether higher levels of these structural protein(s) more effectively support the LGG tumour microenvironment.

We demonstrate that high *DMD* expression is coupled with a significant down-regulation of ribosome pathways including biogenesis and that the DEGs are enriched in biological processes relating to development and morphogenesis. Malignant gliomas recapitulate steps in neurodevelopment to form organ-like structures^27^ and high *DMD* expression may support such a strategy. *TP53* mutation and *ATRX* loss are characteristic of IDH mutant, non-1p/19q co-deleted LGG^11^. *TP53* encodes the tumour suppressor p53 which is tightly linked to ribosome activity^28^ and *ATRX* encodes a chromatin remodelling protein essential for development. Thus, high *DMD* expression effects biological pathways relevant to the functions of known LGG biomarkers as well as overall cellular functions related to LGG tumourigenesis. Our analysis does not implicate a single *DMD* gene product but rather we observed an overall comparable effect of high expression on survival across both full length and short *DMD* gene products. There were however some differences in the DEGs and pathway analyses between the individual gene products, namely there was a more widespread transcriptomic response when Dp71ab and Dp427m were highly expressed than observed for high Dp71 or overall *DMD* expression. Dp427m expression was also the only gene product associated with an alteration of biological process related to the immune system.

Overall, our data identifies *DMD* expression as an independent prognostic marker for LGG and highlights a potentially important role for *DMD* gene product(s) in the progression of low-grade glioma. This knowledge may help reduce and manage the unpredictable nature of LGG progression and recurrence by improving risk stratification.

## Methods

### RNAseq and clinical datasets

The Cancer Genome Atlas (TCGA) LGG dataset and corresponding clinical data was downloaded from cBioPortal after querying for the *DMD* gene (Brain Lower Grade Glioma, TCGA, PanCancer Atlas 2018). The data extracted was mRNA expression, RSEM (batch normalized from Illumina HiSeq_RNASeqV2). The replication dataset was downloaded from the Chinese Glioma Genome Atlas (CGGA), dataset ID: mRNAseq_693. The Firebrowse portal was used to extract TCGA RNAseq isoform data from the LGG mRNASeq archives (illuminahiseq_rnaseqv2-RSEM_isoforms_normalized MD5) and the case IDs matched to those obtained from cBioPortal. *DMD* transcript IDs were matched to specific transcripts using the table browser tool from the UCSC genome browser. We note however, that the transcript expression estimates by RSEM may not be 100% accurate. Note the TCGA PanCancer Atlas 2018 dataset downloaded from cBioPortal contains anaplastic grade III cases which are identifiable by cross matching with clinical data from Firebrowse. Unless otherwise stated, only the grade II (non-anaplastic) cases were used in our analyses.

### Cut-point selection and survival analysis

X-tile (version 3.6.1, Yale University 2003–2005^29^) was used to dichotomise the datasets into high and low *DMD* (or individual gene product) expression groups using a minimal P-value approach. The optimal cut-point value (defined as the brightest pixel on the X-tile plot of chi-squared log-rank values) generated by X-tile was used for survival analysis. A cut-point was generated for each gene product and used across all tumour subtypes. The cut-point used for the TCGA total *DMD* expression dataset was 1183.5 RSEM. The cut-points for Dp427m, Dp71, Dp71a, Dp71b and Dp71ab were 312.1, 278.6, 465.5, 0.01 and 336.2 RSEM respectfully. The cut-point for the CGGA total *DMD* expression dataset was 8.0 FPKM. Plots showing the variability of low versus high expression are presented in Supplementary Fig. S8. Kaplan–Meier curves were analysed using the log-rank test in GraphPad and multivariate Cox regression analysis was conducted in SPSS. Age at diagnosis data was split into young and old groups using the mean (41 years). Unless otherwise stated, significance was set at 0.05 and asterisks used to indicate the level of significance: *P = ≤ 0.05, **P = ≤ 0.01, ***P = ≤ 0.001 and ****P = ≤ 0.0001. For multiple pairwise comparisons the alpha value was set at 0.017 correcting only for the comparisons that were planned (three high vs low groups).

### Identification of differentially expressed genes (DEGs)

We used the integrated web application, iDEP 0.93^30^, hosted at http://ge-lab.org/idep/ for data pre-processing and log transformation of normalised expression values. The full genomic TCGA data was downloaded from cBioPortal, case IDs were matched to the *DMD* (or *DMD* gene product) expression data and formatted into low and high groups using the cut-points derived from X-tile. iDEP encompasses many R packages for bioinformatic analysis of RNAseq data. DEGs were identified using the DESeq2 method within iDEP (false discovery rate (FDR) cut-off of 0.05 and a minimum fold-change of 2). Heatmaps, volcano and M-A plots were generated in iDEP. Functional enrichment analysis of DEGs was performed in iDEP using gene ontology (GO) biological processes, enrichment trees and networks were generated in iDEP.

### Protein–protein interaction analysis

The online Search Tool for the Retrieval of Interacting Genes (STRING) was used to identify the protein–protein interaction (PPI) network connectivity of DEGs. The minimum required interaction score was set to median confidence at 0.4. The resulting network files were imported to Cytoscape v3.8.2 and analysed using the Cytohubba plugin. The top 20 nodes were ranked by the Maximal Clique Centrality (MCC) and Density of Maximum Neighbourhood Component (DMNC) algorithms and the extended subnetwork displayed. Venn diagrams were created using an online tool at http://bioinformatics.psb.ugent.be/webtools/Venn/.

### Pathway analysis

Pathway analysis for high-low comparisons was performed in iDEP using the generally applicable gene set enrichment (GAGE) method and the genes were annotated according to GO biological processes and Kyoto Encyclopaedia of Genes and Genomes (KEGG). The minimum and maximum gene set sizes were set to 15 and 2000 respectively, the pathway significance cut-off (FDR) was set to 0.2, and the top 30 pathways were retrieved. Pathway trees were created in iDEP.

### Co-expression network construction and functional enrichment

Weighted gene co-expression network analysis (WGCNA) was performed in iDEP. The top 1000 variable genes were included with a soft threshold of five and a minimum module size of 20 genes. Module networks of the top 20 genes in each module were created in the network tab of iDEP using an edge threshold of 0.4. Functional enrichment analysis was performed using GO biological processes in iDEP and the resulting enriched pathway tables for each module were exported. The adjusted P values were converted to -log10 and a heatmap created in GraphPad.

### Analysis of regional variations of genomic features

Position RElated Data Analysis (PREDA) was conducted within iDEP using a minimum FDR of 0.01 and minimum statistic of 0.5.

### Immunohistochemistry

Haematoxylin and eosin (H&E) and unstained formalin-fixed paraffin-embedded (FFPE) sections from 24 LGG cases were obtained from University Hospital Southampton NHS Foundation Trust as part of BRAIN UK and under the extended ethical approval of the South Central—Hampshire B Research Ethics Committee (REC reference: 19/SC/027, IRAS project ID: 262890). The cohort included 18 cases of astrocytoma, one case of oligodendroglioma and five cases of ‘not otherwise specified’. Nine out of the 24 cases were IDH mutant and 15 were IDH wild-type as previously determined by the providing centre. Sections were deparaffinised with xylene and rehydrated through graded alcohol. Antigen retrieval was achieved by immersing sections in pH 6.0 citrate buffer and heating in a 800 W microwave for 10 min at high power and 10 min at low power. Immunohistochemistry was performed using a C-terminal anti-dystrophin antibody (Abcam 15277) at a 1:100 dilution followed by a Tyramide SuperBoost™ kit (Invitrogen) according to manufactures’ instructions. Staining was visualised using 3,3′-diaminobenzidine tetra hydrochloride (DAB), and counterstained with haematoxylin.

## Supplementary Information

Dataset S1.

Dataset S2.

Supplementary Information.

## Data Availability

All datasets underpinning this publication are openly available from the University of Northampton Research Explorer at 10.24339/ae20690c-1bdf-48b0-8c5d-c88fbc40ad4a.
