# Supplementary material for: Duchenne muscular dystrophy gene expression is an independent prognostic marker for IDH mutant low-grade glioma
Source: Sci Rep. 2022 Feb 25;12:3200. doi: 10.1038/s41598-022-07223-2 (PMC8881458; doi:10.1038/s41598-022-07223-2)
Supplement: Supplementary file 3 — Supplementary Information. [file 41598_2022_7223_MOESM3_ESM.docx]

|  | B | SE | Wald | df | Sig. | Exp(B) | 95.0% CI for Exp(B) | |
| --- | --- | --- | --- | --- | --- | --- | --- | --- |
|  |  |  |  |  |  |  | Lower | Upper |
| DMD low=0; high=1 | 0.892 | 0.316 | 7.955 | 1 | **0.005** | 2.441 | 1.313 | 4.539 |
| Tumour subtype |  |  | 4.426 | 2 | 0.109 |  |  |  |
| Oligodendroglioma vs. astrocytoma | 0.027 | 0.439 | 0.004 | 1 | 0.951 | 1.028 | 0.435 | 2.427 |
| NOS vs. astrocytoma | 0.638 | 0.416 | 2.353 | 1 | 0.125 | 1.893 | 0.838 | 4.279 |
| Age at diagnosis | 0.562 | 0.322 | 3.045 | 1 | 0.081 | 1.755 | 0.933 | 3.299 |
| IDH mutation status | -2.163 | 0.381 | 32.300 | 1 | **<0.001** | 0.115 | 0.055 | 0.242 |

**Supplementary table S1. SPSS output for multivariate Cox regression analysis of *DMD* expression with tumour subtype**

**Supplementary figure S1. *DMD* expression is only significantly associated with survival in lower-grade gliomas.** TCGA RNAseq data from WHO LGG grade II, III or IV was dichotomised into high (blue) and low (red) *DMD* expressing groups and survival analysis performed in GraphPad using the log-rank test. Numbers in brackets are median overall survival times in months.

| **Cohort** | **Grade II** | **Grade III** | **Grade IV** |
| --- | --- | --- | --- |
| **TCGA** | **✓** | × | × |
| **CGGA** | × | **✓** | × |

**Supplementary figure S2. A replication cohort analysis confirms the detrimental effect of high *DMD* expression on glioma survival is limited to the less invasive gliomas.** CGGA RNAseq data from grade II, III or IV LGG cases was dichotomised into high (blue) and low (red) *DMD* expressing groups and survival analysis performed in GraphPad using the log-rank test. Numbers in brackets are median overall survival times in months. The table highlights for which tumour grade *DMD* expression was significant for both TCGA and CGGA cohorts.

a

|  | B | SE | Wald | df | Sig. | Exp(B) | 95.0% CI for Exp(B) | |
| --- | --- | --- | --- | --- | --- | --- | --- | --- |
|  |  |  |  |  |  |  | Lower | Upper |
| Age at diagnosis 0=<41; 1=>41 | 0.494 | 0.309 | 2.552 | 1 | 0.11 | 1.638 | 0.894 | 3.001 |
| IDH mutation status wt=0; mutant co-del=1; mutant non-co-del=2 |  |  | 35.888 | 2 | **<.001 (1.6105E-8)** |  |  |  |
| IDH mutation status wt=0; mutant co-del=1; mutant non-co-del=2(1) | -2.785 | 0.523 | 28.37 | 1 | **<.001 (1.002E-7)** | 0.062 | 0.022 | 0.172 |
| IDH mutation status wt=0; mutant co-del=1; mutant non-co-del=2(2) | -1.985 | 0.377 | 27.778 | 1 | **<.001 (1.3609E-7)** | 0.137 | 0.066 | 0.287 |
| DMD low=0; high=1 | 0.752 | 0.323 | 5.412 | 1 | **0.02** | 2.12 | 1.126 | 3.994 |

b

**Supplementary figure S3. *DMD* expression remains significantly associated with survival in a multivariate model containing important clinicopathological variables.** (a) Age at diagnosis and IDH mutation status are both significantly associated with survival in the TCGA LGG grade II cohort using the log-rank test. Numbers in brackets are median overall survival times in months. (b) *DMD* expression remained significant in a multivariate Cox regression analysis with age (<41 years; >41 years) and IDH mutation/co-deletion status (wild-type; IDH mutant co-deleted and IDH mutant non-co-deleted) and is therefore independently prognostic of these for LGG. Multivariate analysis was conducted using SPSS.

**Supplementary figure S4. A replication cohort analysis confirms the detrimental effect of high *DMD* expression on glioma survival is limited to the less invasive, IDH mutant, gliomas.** CGGA RNAseq data from grade III LGG cases was dichotomised into high (blue) and low (red) *DMD* expressing groups and analysed according to IDH mutation status. Survival analysis was performed in GraphPad using the log-rank test. This data replicates our finding that high *DMD* expression is significantly linked to poor survival in only IDH mutant cases. Numbers in brackets are median overall survival times in months.

**Supplementary table S2. DEGs present in WGCNA modules.**

| **Names** | **Total** | **Genes** |
| --- | --- | --- |
| **DEGs present in turquoise module** | 10 | NDST3 |
|  |  | TMPRSS3 |
|  |  | EPHA6 |
|  |  | SLC39A12 |
|  |  | SP8 |
|  |  | CDKL5 |
|  |  | VGF |
|  |  | WIF1 |
|  |  | GABRA2 |
|  |  | SLC7A10 |
| **DEGs present in blue module** | 44 | CREB3L1 |
|  |  | ZNF676 |
|  |  | MOXD1 |
|  |  | LEFTY2 |
|  |  | TRIM67 |
|  |  | HOXA13 |
|  |  | HCG22 |
|  |  | COL28A1 |
|  |  | DUSP4 |
|  |  | SEMA3A |
|  |  | AMH |
|  |  | SHISA9 |
|  |  | BARHL1 |
|  |  | POU4F1 |
|  |  | GALR1 |
|  |  | LHX5 |
|  |  | MSX2 |
|  |  | TFPI |
|  |  | TRPM3 |
|  |  | MSTN |
|  |  | RPE65 |
|  |  | CDH7 |
|  |  | SULF1 |
|  |  | PDGFD |
|  |  | MADCAM1 |
|  |  | ETV4 |
|  |  | C10orf105 |
|  |  | STC2 |
|  |  | OR4N2 |
|  |  | LRRC14B |
|  |  | COL11A1 |
|  |  | CLEC4F |
|  |  | DDIT4L |
|  |  | ACAN |
|  |  | RELN |
|  |  | EMILIN3 |
|  |  | AR |
|  |  | MMEL1 |
|  |  | PCDHGA3 |
|  |  | ZFR2 |
|  |  | GDF8 |
|  |  | ACY3 |
|  |  | TNNT1 |
|  |  | LHX1 |
| **DEGs present in green module** | 1 | IL1B |

a


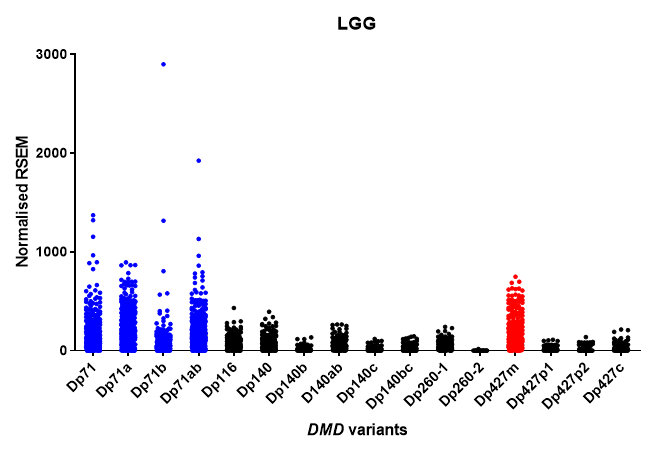


b

**Supplementary figure S5. The four Dp71 isoforms and Dp427m are the most predominant *DMD* gene products expressed in LGG tissue.** (a) RNAseq isoform data was extracted from the TCGA cohort and the expression level (normalised RSEM) for each *DMD* gene product per sample was plotted. (b) Scatter graphs for Spearman r correlation analysis of each gene product against total *DMD* gene expression. The correlation matrix represents r values for all correlation combinations.

**Supplementary figure S6. Tumour subtype analysis for each *DMD* gene product confirms that the expression of multiple *DMD* gene products are associated with LGG survival across all subtypes.** The TCGA LGG cohort was divided into tumour subtype groups, each group was further dichotomised into high (blue) or low (red) *DMD* expressing groups. Survival analysis was performed in GraphPad using the log-rank test. The alpha value was adjusted to 0.017 to correct for multiple testing (five separate analyses; three tests per gene product), bold P values indicate significance. A: astrocytoma; OD: oligodendroglioma; NOS: not otherwise specified. Numbers in brackets are median overall survival times in months.

**Supplementary table S3. SPSS output for multivariate Cox regression analysis of Dp71**

|  | B | SE | Wald | df | Sig. | Exp(B) | 95.0% CI for Exp(B) | |
| --- | --- | --- | --- | --- | --- | --- | --- | --- |
|  |  |  |  |  |  |  | Lower | Upper |
| Age at diagnosis 0=<41; 1=>41 | 0.547 | 0.314 | 3.029 | 1 | 0.082 | 1.728 | 0.933 | 3.2 |
| IDH mutation status wt=0; mutant co-del=1; mutant non-co-del=2 |  |  | 37.025 | 2 | **<.001 (9.1225E-9)** |  |  |  |
| IDH mutation status wt=0; mutant co-del=1; mutant non-co-del=2(1) | -2.924 | 0.511 | 32.691 | 1 | **<.001 (1.0803E-8)** | 0.054 | 0.02 | 0.146 |
| IDH mutation status wt=0; mutant co-del=1; mutant non-co-del=2(2) | -1.957 | 0.389 | 25.263 | 1 | **<.001 (5.002E-7)** | 0.141 | 0.066 | 0.303 |
| Dp71 low=0; high=1 | 0.523 | 0.362 | 2.081 | 1 | 0.149 | 1.687 | 0.829 | 3.432 |

**Supplementary table S4. SPSS output for multivariate Cox regression analysis of Dp71a**

|  | B | SE | Wald | df | Sig. | Exp(B) | 95.0% CI for Exp(B) | |
| --- | --- | --- | --- | --- | --- | --- | --- | --- |
|  |  |  |  |  |  |  | Lower | Upper |
| Age at diagnosis 0=<41; 1=>41 | 0.587 | 0.308 | 3.638 | 1 | 0.056 | 1.799 | 0.984 | 3.289 |
| IDH mutation status wt=0; mutant co-del=1; mutant non-co-del=2 |  |  | 40.335 | 2 | **<.001 (1.7435E-9)** |  |  |  |
| IDH mutation status wt=0; mutant co-del=1; mutant non-co-del=2(1) | -3.011 | 0.51 | 34.842 | 1 | **<.001 (3.5762E-9)** | 0.049 | 0.018 | 0.134 |
| IDH mutation status wt=0; mutant co-del=1; mutant non-co-del=2(2) | -2.035 | 0.383 | 28.201 | 1 | **<.001 (1.0932E-7)** | 0.131 | 0.062 | 0.277 |
| Dp71a low=0; high=1 | 0.133 | 0.405 | 0.108 | 1 | 0.742 | 1.142 | 0.517 | 2.525 |

**Supplementary table S5. SPSS output for multivariate Cox regression analysis of Dp71ab**

|  | B | SE | Wald | df | Sig. | Exp(B) | 95.0% CI for Exp(B) | |
| --- | --- | --- | --- | --- | --- | --- | --- | --- |
|  |  |  |  |  |  |  | Lower | Upper |
| Age at diagnosis 0=<41; 1=>41 | 0.51 | 0.316 | 2.595 | 1 | 0.107 | 1.665 | 0.895 | 3.095 |
| IDH mutation status wt=0; mutant co-del=1; mutant non-co-del=2 |  |  | 37.613 | 2 | **<.001 (6.8004E-9)** |  |  |  |
| IDH mutation status wt=0; mutant co-del=1; mutant non-co-del=2(1) | -2.919 | 0.513 | 32.446 | 1 | **<.001 (1.2256E-8)** | 0.054 | 0.02 | 0.147 |
| IDH mutation status wt=0; mutant co-del=1; mutant non-co-del=2(2) | -1.987 | 0.386 | 26.569 | 1 | **<.001 (2.5435E-7)** | 0.137 | 0.064 | 0.292 |
| Dp71ab low=0; high=1 | 0.66 | 0.394 | 2.807 | 1 | 0.094 | 1.936 | 0.894 | 4.191 |

**Supplementary table S6. SPSS output for multivariate Cox regression analysis of Dp427m**

|  | B | SE | Wald | df | Sig. | Exp(B) | 95.0% CI for Exp(B) | |
| --- | --- | --- | --- | --- | --- | --- | --- | --- |
|  |  |  |  |  |  |  | Lower | Upper |
| Age at diagnosis 0=<41; 1=>41 | 0.477 | 0.314 | 2.302 | 1 | 0.129 | 1.611 | 0.87 | 2.981 |
| IDH mutation status wt=0; mutant co-del=1; mutant non-co-del=2 |  |  | 44.151 | 2 | **<.001 (2.586E-10)** |  |  |  |
| IDH mutation status wt=0; mutant co-del=1; mutant non-co-del=2(1) | -3.054 | 0.505 | 36.552 | 1 | **<.001 (1.4868E-9)** | 0.047 | 0.018 | 0.127 |
| IDH mutation status wt=0; mutant co-del=1; mutant non-co-del=2(2) | -2.209 | 0.393 | 31.671 | 1 | **<.001 (1.8259E-8)** | 0.11 | 0.051 | 0.237 |
| Dp427m low=0; high=1 | 0.67 | 0.386 | 3.008 | 1 | 0.083 | 1.954 | 0.917 | 4.165 |

**Supplementary table S7. Identified hub genes amongst the DEG PPI networks for high vs low Dp71, Dp71ab or Dp427m expression**

|  | **Dp71** | **Dp71ab** | **Dp427m** |
| --- | --- | --- | --- |
| MCC | PGF, TLX1, AQP4 | SYNPR, GRIN1, SLC6A7, VSTM2B, SYN1, HTR5A, FAM163B, SNCB, MAG, SCRT1, LY6H, CCK, GABRG2, SNAP25, GABRD, SLC32A1, CA10 | NGB, SYNPR, GRIN1, SYN1, HTR5A, SNCB, HPCA, SCRT1, CPNE6, HRH3, CCK, OPALIN, GABRG2, SNAP25, GABRD, SLC32A1, ATP2B3 |
| DMNC | TGFB2, MFAP5, SLC2A10 | KIF20A, ESPL1, E2F7, CENPA, CDCA2, KIF14, ASPM, BUB1B, DLGAP5, KIF4A, KIF18A, KIF23, DEPDC1, SLC8A2, TMEM151B, CDCA8, KIF2C | KIF20A, CENPF, CDCA2, P2RY12, KIF14, ASPM, GRM7, CENPI, SOHLH1, PRR11, S1PR3, CLSPN, DLGAP5, KIF18A, TTK, DEPDC1, TMEM151B |
| Common | GPR143, NPB, GJB6, RYR3, TMPRSS3, NDST3, NDST4, SDC4, SLC26A4, TMC1, P2RY2, GNG12, GPR17, GALR1, HS6ST3, GJA1, GPC5 | CENPF, CALY, PHYHIP | SLC6A, CALY, PHYHIP |

MCC: Maximal Clique Centrality; DMNC: Density of Maximum Neighbourhood Component. Common: hub genes returned with both MCC and DMNC algorithms.


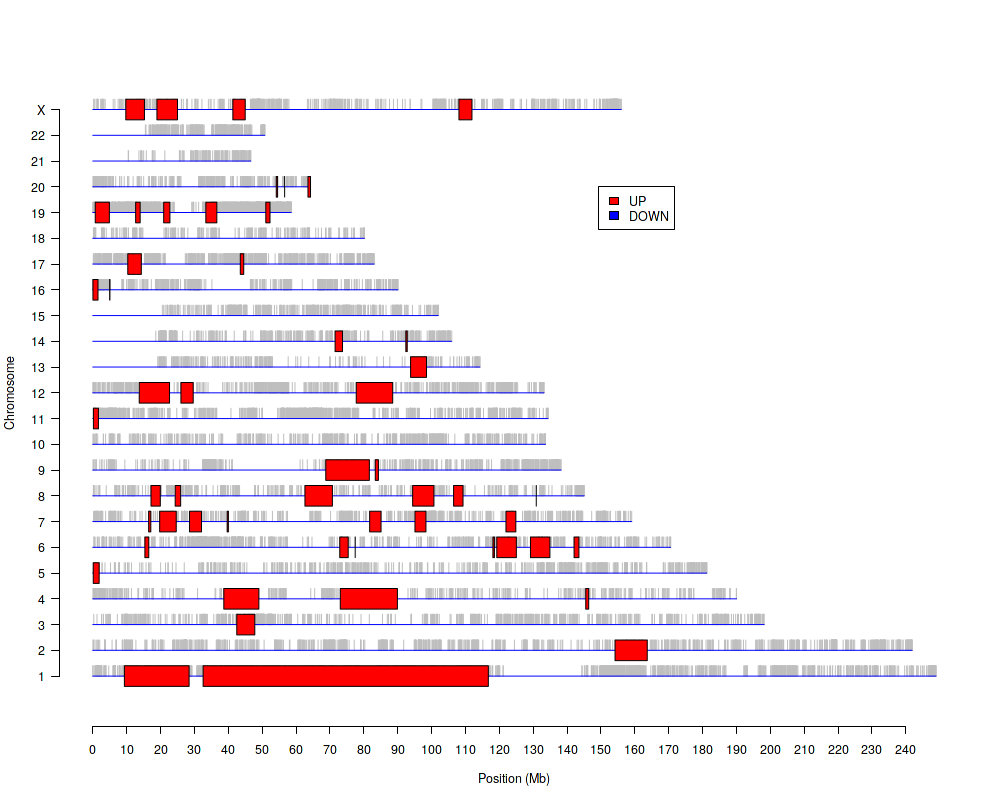
a

Dp71


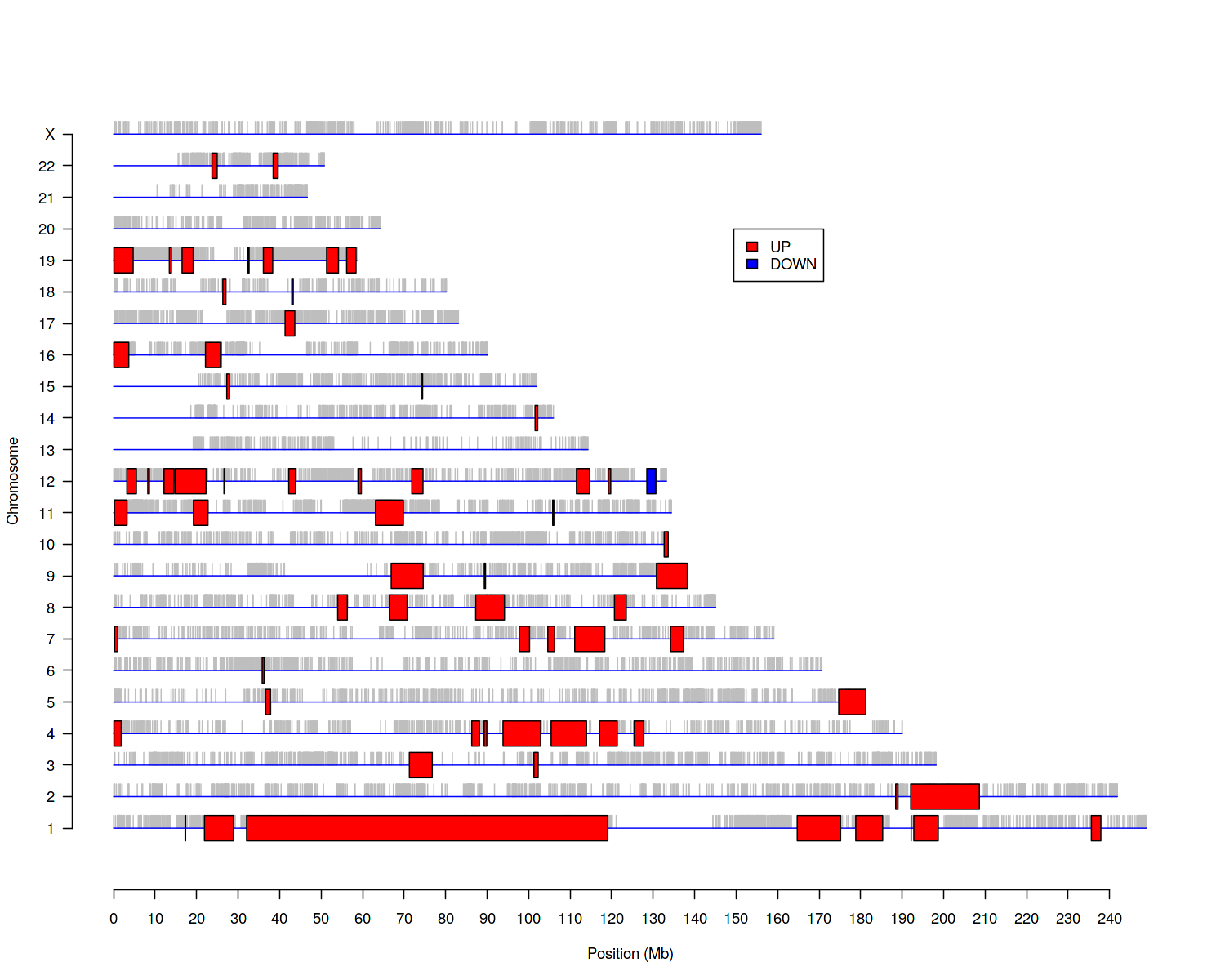
b

Dp427m

**Supplementary figure S7. PREDA reveals genomic regions significantly altered upon high vs low *DMD* gene product expression.** PREDA was performed in iDEP for (a) high vs low Dp71 and (b) Dp427m. No significant regions were found using PREDA with the high vs low Dp71ab dataset.

**Supplementary figure S8. Plots to illustrate the variability of dystrophin expression across both low and high expression groups.** Data is shown for total *DMD* expression in both TCGA and CGGA cohorts. *DMD* transcript data is available for the TCGA dataset only.

**Supplementary data S1.** Complete list of identified DEGs for high versus low *DMD*, Dp71, Dp71ab and Dp427m expression in LGG. Provided as a separate xlsx. file.

**Supplementary data S2.** Complete list of genes identified by WGCNA that are correlated in all LGG samples. Module number and colour for each gene are indicated. Provided as a separate xlsx. file.
